# Risk assessment for vaccination programmes in a pandemic. A Swedish model for efficacy and safety during COVID-19

**DOI:** 10.48101/ujms.v130.13602

**Published:** 2025-11-24

**Authors:** Gerhard Wikstöm, Michael Welsh

Risk assessment of vaccination programmes is of paramount importance for public trust in vaccines. The recent coronavirus disease 2019 (COVID-19) pandemic illustrates this dilemma. In order to achieve group immunisation, there was a need for rapid development of effective vaccines that could be quickly administered to the general population. However, quality could not be compromised. Thus, the follow-up of these vaccination programmes demanded a strict control of the properties of the vaccine types and of the manufacturers’ routines, especially if the vaccines were of a new type that previously had not been used.

The nationwide register-based study cohort created by the Swedish Medical Products Agency with regular updates of individual level linkage of COVID-19 vaccination exposure data to other health data registers facilitated both safety signal detection and evaluation and other pharmacoepidemiological studies.

Several COVID-19 vaccines showed a demonstrated efficacy as high as 95% in preventing symptomatic COVID-19 infections. Already in June 2021, two mRNA vaccines (Comirnaty® and Spikevax®) and two viral vector vaccines (Vaxzevria® and COVID-19 Vaccine Janssen) were granted marketing authorisation in the European Union (EU). Several other vaccines from that time were under evaluation for marketing approval. This allowed information on dates of licensing and relevant safety aspects of importance identified by the European Medicines Agency (EMA) to be presented in a chronological order. For Swedish circumstances, this information is presented in a paper by Zethelius et al. (1).

In addition to the regular pharmacovigilance routines in place for all medicines, the Swedish Medical Products Agency initiated a project for epidemiological surveillance to detect and characterise suspected adverse effects of COVID-19 vaccines in Sweden. This decision and study set-up was partly based on the study findings that narcolepsy (2) was a side effect which was reported following the nationwide vaccination campaign during the 2009–2010 H1N1 influenza pandemic.

The COVID-19 vaccines were rapidly rolled out in national vaccination programmes worldwide after accelerated approval processes. The large population exposure achieved in a very short time required systematic monitoring of all safety aspects. A meticulous pharmacovigilance in Sweden was thus able to detect and characterise suspected adverse effects of COVID-19 vaccines, the methodology and results of which are presented in a recent paper published in Uppsala Journal of Medical Sciences (1). This information can be used to increase knowledge on how quickly routines in situations like the COVID-19 pandemic, displaying initially lethal effects on the population, can be implemented.

The primary study cohort consisted of 8,305,978 adults who were 18 years and older and permanently residing in Sweden as on 31 December 2020. National vaccination campaigns were then launched in a phased manner during 2021. The elderly, people with chronic diseases and nursing home personnel were prioritised during primary vaccinations and for initial boosters. In the Swedish population, 85% of those above the age of 12 received at least one vaccine dose. Moreover, 81% of those above the age of 18 and 95% of those above the age of 80 received at least two doses.

Monthly safety reviews performed by the EMA identified risk for thrombosis with thrombocytopenia syndrome with adenoviral vaccines and myocarditis for mRNA vaccines led to restrictions in national recommendations for high-risk groups.

The vaccine coverage was high. Timelines presented should be considered in follow-up studies of COVID-19 vaccines to manage possible selection bias and confounding factors.

In conclusion, the proactive decisions made by the Swedish Medical Products Agency in 2020 to make a register with increased pharmacovigilance qualities seem to have increased the safety feature in the handling of a vaccination programme with good coverage and allowed relevant adverse drug reactions to be identified (3). Hopefully, dissemination of the conclusions from that study among the general population will increase the acceptance for vaccinations whenever warranted in the future.


Professor Gerhard Wikstöm
Professor Michael Welsh Editors

